# Role of CTLA4 in the Proliferation and Survival of Chronic Lymphocytic Leukemia

**DOI:** 10.1371/journal.pone.0070352

**Published:** 2013-08-01

**Authors:** Amit K. Mittal, Nagendra K. Chaturvedi, Rae A. Rohlfsen, Payal Gupta, Avadhut D. Joshi, Ganapati V. Hegde, R. Gregory Bociek, Shantaram S. Joshi

**Affiliations:** 1 Department of Genetics, Cell Biology and Anatomy, University of Nebraska Medical Center, Omaha, Nebraska, United States of America; 2 Internal Medicine, Section of Oncology/Hematology, University of Nebraska Medical Center, Omaha, Nebraska, United States of America; University of Oslo, Norway

## Abstract

Earlier, we reported that CTLA4 expression is inversely correlated with CD38 expression in chronic lymphocytic leukemia (CLL) cells. However, the specific role of CTLA4 in CLL pathogenesis remains unknown. Therefore, to elucidate the possible role of CTLA4 in CLL pathogenesis, CTLA4 was down-regulated in primary CLL cells. We then evaluated proliferation/survival in these cells using MTT, ^3^H-thymidine uptake and Annexin-V apoptosis assays. We also measured expression levels of downstream molecules involved in B-cell proliferation/survival signaling including STAT1, NFATC2, c-Fos, c-Myc, and Bcl-2 using microarray, PCR, western blotting analyses, and a stromal cell culture system. CLL cells with CTLA4 down-regulation demonstrated a significant increase in proliferation and survival along with an increased expression of STAT1, STAT1 phosphorylation, NFATC2, c-Fos phosphorylation, c-Myc, Ki-67 and Bcl-2 molecules. In addition, compared to controls, the CTLA4-downregulated CLL cells showed a decreased frequency of apoptosis, which also correlated with increased expression of Bcl-2. Interestingly, CLL cells from lymph node and CLL cells co-cultured on stroma expressed lower levels of CTLA4 and higher levels of c-Fos, c-Myc, and Bcl-2 compared to CLL control cells. These results indicate that microenvironment-controlled-CTLA4 expression mediates proliferation/survival of CLL cells by regulating the expression/activation of STAT1, NFATC2, c-Fos, c-Myc, and/or Bcl-2.

## Introduction

Chronic lymphocytic leukemia (CLL), a very heterogenous disease with a variable clinical course, is the most common adult leukemia in the western world [1]. CLL is characterized by an abnormal accumulation of monoclonal and mature CD5^+^ CD19^+^ CD23^+^ B-cells in the peripheral blood, bone marrow, and lymph nodes [2]. Prognostic markers such as the status of immunoglobulin VH gene (IgVH) mutations, chromosomal abnormalities, CD38 expression, and ZAP-70 expression have been useful in predicting the clinical outcome in CLL [3]–[5].

CD38 is a 45 kDa transmembrane glycoprotein, which appears to utilize the B cell antigen receptor (BCR) signaling pathway to induce survival and proliferation in CLL cells [6]. We and others have shown that cytotoxic T-lymphocyte antigen 4 (CTLA4) is overexpressed in low CD38-expressing CLL clones compared to high CD38-expressing CLL clones [5], [7]. In addition, CTLA4 reliably predicted the clinical outcome of CLL patients; higher expression of CTLA4 is associated with good clinical outcome [5]. Moreover, the presence of a polymorphism of CTLA4 has been correlated to increased risk and advanced Rai stages in CLL [8].

Aberrant expression of co-stimulatory molecules and co-inhibitory molecules can increase or decrease the risk of cancer. CTLA4 is mainly expressed on CD4+ T cells. It is a member of the CD28 receptor family that shares many features with CD28 including a gene locus on chromosome 2q33-34, a single disulfide-linked extracellular IgV-like domain, and the tendency to function as a dimer [9]. CTLA4 binds to the CD80 (B7-1) and CD86 (B7-2) ligands found on B-cells, but unlike the CD28 receptor, its much higher affinity for CD80 inhibits secondary activation of T-cells by inhibiting the phosphorylation of Akt [10], [11]. In addition, it has been shown that CTLA4 can inhibit cell cycle progression in T-cells by inhibiting production of cyclin D3 and cyclin-dependent kinases [12]. By contrast, T-cells show an increase in activation and proliferation in the absence of CTLA4 [13].

Previous studies reported higher expression of CTLA4 in T-cells from CLL patients compared to healthy donors. Moreover, T-cells co-cultured with activated CLL cells showed higher proliferation when CTLA4 was blocked using anti-CTLA4 antibodies [14]. Expression of CTLA4 was also higher on leukemic B-cells than on its normal counterpart. Furthermore, CTLA4 expression was associated with a higher number of CLL cells in G0–G1 phase, indicating that CTLA4 may delay cell cycle progression [15].

CTLA4 has been shown to be a promising target for the treatment of many chronic immunological and autoimmune diseases [16]–[18]. Together, these findings warrant further study of CTLA4 to elucidate its role in the proliferation/survival of CLL cells. Therefore, we hypothesized that CTLA4 inhibits CLL cell proliferation/survival by regulating the downstream molecules of the B-cell proliferation/survival signaling pathway. In the present study we have shown that downregulation of CTLA4 in CLL cells increases their proliferation/survival and increases expression of STAT1, NFATC2, c-Fos, c-Myc, and Bcl-2. These molecules are known to increase the proliferation/survival of cells, indicating that CTLA4 might inhibit the proliferation/survival of CLL cells via downregulating the expression of these molecules. Thus, this study suggests a molecular mechanism by which CTLA4 controls proliferation/survival of CLL cells.

## Materials and Methods

### Ethics Statement

CLL samples were collected from 105 CLL patients at the University of Nebraska Medical Center (UNMC) clinic/hospital. For the collection of these samples a protocol approved by the UNMC Institutional Review Board (IRB) was used. Before collecting the CLL sample, the participants were provided with a written consent form containing the details of the study and approved by the UNMC IRB. The blood was collected only from the patients who consented by signing the consent form.

### Blood Collection and Isolation of CLL Cells

Peripheral blood (PB) samples were collected from 105 CLL patients with informed consent using an Institutional Review Board-approved protocol. Only untreated CLL patients or patients who had not received treatment in the past 6 months were included in this study. The patient characteristics are described in Table S1. CLL cells were isolated from whole blood by centrifugation using lymphocyte separation medium (Accurate Chemical and Scientific Corp., NY). The purity and immunophenotype of the isolated CLL cells were then determined by flow cytometry. Additional CLL samples from PB (n = 20), bone marrow (BM) (n = 18), and lymph nodes (LN) (n = 15) were also isolated to study the influence of the microenvironment on the expression of selected signaling molecules. In brief, CLL cells from PB and BM were isolated using the same protocol described above. CLL cells from frozen LN were isolated using a combination of immunohistochemistry and Laser microdissection techniques as described earlier [19].

### Flow Cytometry

To determine the immunophenotypes of CLL cells, flow cytometry was performed using the following combinations of antibodies: CD3-FITC and CD19-PE, and CD5-PE and CD19-FITC, and CD38-PE and CD19-FITC, and CD19-FITC and CTLA4-APC, and CD19-FITC and Ki-67-PE (BD Biosciences, CA). CLL cells (2×10^5^) were stained with 5 µl of fluorochrome-labeled antibodies, and the percentage of positive cells for each marker was determined using a Becton Dickinson FACStar-plus flow cytometer (BD Biosciences, CA). Samples containing more than 90% CLL cells were used for this study. Samples with more than 30% CD38+ CLL cells were grouped into the high-CD38 group, while samples containing less than 30% CD38+ CLL cells were grouped into the low-CD38 group. Our definition of high CD38 CLL are CD5+, CD19+, and over 30% CD38 positive cells. Similarly, low CD38 cells are CD5+, CD19+, and less than 30% CD38 expressing cells. Our selection of 30% cutoff for CD38 expression is based on the majority of the literature [3], [5]–[7], [20].

### Cytogenetic Analyses

Fluorescent *in situ* hybridization (FISH) was performed by the Human Genetics Institute at the University of Nebraska Medical Center to identify cytogenetic abnormalities in CLL cells from patients as previously described [21], [22]. Those with chromosome 11q deletion, 17p deletion, and trisomy 12 were classified as the poor-outcome group, while those with a normal karyotype and 13q deletion were grouped as the good-outcome group.

### Downregulation of CTLA4 in CLL Cells Using Antisense Oligonucleotide and siRNA

CTLA4 was downregulated in CLL cells using a 5 µM concentration of a CTLA4 antisense (AS) oligonucleotide (ATGGCTTGCCTTGGATTTCA). An irrelevant AS oligonucleotide (TATGCTGTGCCGGGGTCTTCGGGC) was used as a control. The CLL cells were incubated with the CTLA4 AS for 24, 48, and 72 hours, and CTLA4-downregulated CLL cells were used in different assays described below. CTLA4 was also down-regulated by transient transfection of 100 nM CTLA4-siRNA (Santacruz Biotechnology) using Lipofectamine™ (Invitrogen, CA).

### Cell Proliferation

CLL cell proliferation was measured in the presence of CTLA4 AS or irrelevant AS by both MTT and ^3^H-thymidine uptake assays. Purified CLL cells (5×10^4^ cells/well) were plated in 96-well plates and triplicate wells were tested after incubation with AS for 24, 48, and 72 hours. For the MTT assays, MTT reagent was added 2 hours prior to the end of incubation, and MTT lysis buffer was added at the end of incubation. For the ^3^H-thymidine uptake assays, ^3^H-thymidine was added 16–18 hours before the desired cell-harvest time. Cells were harvested using a PHD cell harvester (Cambridge Technology, MA) onto filter paper disks (Brandel Inc., MD). Radioactivity was measured by placing the disk in 1 ml of scintillation fluid using a Packard liquid scintillation counter.

### Isolation of RNA from CLL Cells and cDNA Preparation

Total RNA was extracted from CLL cells using the TRIzol™ (Invitrogen, CA) method according to the manufacturer’s instructions. RNA quantity and purity were determined by UV spectrophotometry and by electrophoresis on a 2% agarose gel. RNA was then reverse transcribed using random hexamer primers and the superscript RT enzyme (Invitrogen, CA).

### Microarray Analysis

Gene expression profiling was performed using a DNA microarray chip (MWG Biotech, Germany, Human 10 K oligo set A) consisting of a 50-mer oligonucleotide representing 10,000 different genes. The RNA from CLL samples and Stratagene™ reference mRNA were reverse transcribed and then labeled with Cy3 or Cy5 fluorescence dyes and hybridized with the array chip as described previously [5]. The hybridized slides were scanned and images were collected by an Axon 4000B scanner (Axon Instruments, CA). The median fluorescence intensity for each spot/gene was obtained using GenePix 6.0 software. Differentially expressed genes between good- and poor-outcome groups were identified using significance analyses of microarray (SAM).

### Semi-quantitative RT-PCR

CTLA4 downregulation by AS after a 24-hour *in vitro* incubation period was confirmed using semiquantitative reverse transcription (RT)-PCR. First-strand cDNA was synthesized as explained above and then amplified using gene-specific forward and reverse primers and Taq polymerase (Invitrogen, CA) in a step-cycle program. PCR products were then visualized on a 2% agarose gel stained with ethidium bromide. The genes involved in the CD38/BCR pathway were identified from previous microarrays reported by our lab [5]; these include NFATC2, STAT1, c-Fos, c-Myc, and Bcl-2. RT-PCR was performed to measure expression of these genes in c-DNA from control CLL cells and from CTLA4-downregulated CLL cells.

### Real-time PCR

SYBR green real-time PCR was used to further confirm the differential expression of STAT1, NFATC2, c-Fos, CTLA4, and c-Myc in high- and low-CD38 subgroups or in control and CTLA4-downregulated CLL cells. The cDNAs were mixed with primers and SYBR-Green PCR Master-mix (Applied Biosystems, CA), and real-time PCR was performed using the ABI Prism-7000 real-time PCR detection system (Applied Biosystems, CA). PCR cycles consisted of 30 seconds at 95°C, 45 seconds at 60°C, and 30 seconds at 72°C. Resulting C_t_ values were used for further analysis. In all PCR reactions, RPL13A and HPRT were used as housekeeping genes to normalize the cDNA quantity. Primers used for each gene are listed in Table S2.

### Western Blotting

To investigate the expression of molecules at the protein level, western blot was performed using anti-human antibodies to CTLA4, STAT1, c-Myc, NFAT1 (Abcam, MA), phospho-STAT1 (Cell Signaling Technology, MA), phospho-c-Fos, c-Fos, Bcl-2, β-Actin and anti-mouse/rabbit-HRP (Santacruz, CA). Western blot analysis was performed using a standardized protocol in the laboratory. Briefly, the cells were harvested after the indicated time, washed with ice-cold PBS and lysed in a RIPA lysis buffer containing protease and phosphatase inhibitor cocktail (Pierce, IL). These protein lysates were subjected to 10–12% SDS-polyacrylamide gel electrophoresis, transferred to PVDF membrane, and then the membrane was blocked with 5% non-fat dry milk and probed with specific primary antibodies. Immunoreactivity was detected using appropriate peroxidase-conjugated secondary antibodies and visualized using ECL detection system (Pierce, IL). The band intensity was measured using Image J software (rsbweb.nih.gov/ij/).

### Annexin–V Apoptosis Assay

To investigate the effect of CTLA4 on CLL cell survival, an Annexin-V assay was performed using apoptosis detection kit (BD Pharmingen, CA). Five million CLL cells were used for each experimental group: untreated CLL cells, CLL cells treated with irrelevant AS, and CLL cells treated with CTLA4 AS. Each group was incubated for 72 hours. Cells were double-stained with Annexin-V-APC and CD19-FITC; flow cytometry was performed to obtain the percentage of apoptotic CLL cells in the experimental groups.

### Co-culture of CLL Cells on Stroma

CLL cells purified from PB were co-cultured on endothelial-derived stromal cells (HMEC) and BM-derived stromal cells as described earlier [23]. CLL cells were co-cultured for 48 to 72 hours on stroma.

### Statistical Analysis

CLL patients were grouped on the basis of CD38 expression and chromosomal abnormalities. Levels of relative gene expression (from real-time PCR) were compared between two prognostic subgroups. Student’s t-test was performed between the groups to determine statistical significance, and a p-value ≤0.05 was considered to be significant.

## Results

### Patients’ Characteristics

The 105 CLL patients were grouped as CD38 high (>30% of their CLL cells expressed CD38) or CD38 low (<30% expressed CD38) on the basis of flow cytometry analysis. There were 37 patients in the high-CD38 group and 68 patients in the low-CD38 group. A significant difference in CD38 expression was observed between these two groups because the median percentage of CD38+ cells in the high-CD38 group was 52% compared to 9% in the low-CD38 group. Multivariant analyses were performed to validate the grouping of patients based on high and low CD38 expression and to identify correlations with other parameters such as age, gender, and other prognostic markers, such as chromosome abnormality, IgVH status, etc. as shown in Table S1. Correlations between high CD38 expression and other prognostic characteristics including unmutated IgVH, chromosomal abnormality, and lymphadenopathy validated our patient groups.

### Effect of CTLA4 Downregulation on CLL Cells

**Figure 1:**
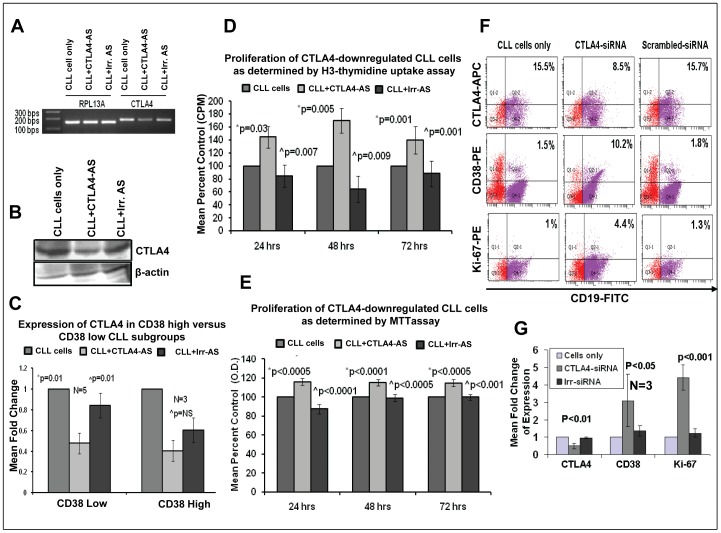
Effects of CTLA4 downregulation on CLL cells. **Panel A:** RT-PCR showing decreased expression of CTLA4 in low CD38/high CTLA4 CLL cells treated with CTLA4 antisense (AS) compared to control CLL cells or CLL cells treated with irrelevant AS. RPL13A was used as a housekeeping gene to normalize the cDNA. **Panel B:** Western blot showing a decrease in the expression of CTLA4 at the protein level in CLL (low CD38/high CTLA4) cells. β-Actin was used as a control. **Panel C:** Real-time PCR results in samples from five different CLL patients with low CD38 and from three different CLL patients with high CD38 confirm CTLA4 downregulation in CLL cells treated with CTLA4 AS compared to control CLL cells or CLL cells treated with irrelevant AS. **Panels D–E:** ^3^H-thymidine uptake assays (Panel D) and MTT assays (Panel E) demonstrating cell proliferation levels in CTLA4-downregulated low CD38/high CTLA4 CLL cells compared to untreated CLL cells and to CLL cells treated with irrelevant AS. *p illustrates the statistical difference between control CLL cells and CTLA4-downregulated CLL cells, while ∧p illustrates the difference between CTLA4-downregulated CLL cells and CLL cells treated with irrelevant AS. **Panel F–G:** Representative results (panel F) of flow cytometry analyses showing the expression of CTLA4, CD38 and Ki-67 among CD19+ gated CLL (low CD38/high CTLA4) cells untreated, treated with scrambled- or CTLA4-siRNA. Panel G showing quantification (fold change of percentage) of flow cytometry analyses results for the expression of CTLA4, CD38 and Ki-67 after downregulation of CTLA4 in three different CLL patient samples.

To determine the role of CTLA4 in the pathogenesis of CLL, CTLA4 expression was downregulated in primary CLL cells from peripheral blood using AS/siRNA. Untreated CLL cells and CLL cells treated with an irrelevant AS or scrambled-siRNA, were used as control. Slight death rate (5–10%) of CLL cells were observed following transfection with siRNA**.** The downregulation of CTLA4 was confirmed first using semi-quantitative RT-PCR, western blot, real-time PCR and flow cytometry as shown in Figure 1A, 1B, 1C, 1F and 1G. The results of semiquantitative RT-PCR demonstrated a significant decrease in the level of CTLA4 transcripts (Figure 1A). Western blot analyses demonstrated a two-fold decrease in CTLA4 protein expression compared to cells treated with irrelevant AS and untreated cells (Figure 1B). To determine the relationship/correlation between CTLA4-downregulation and CD38 expression on CLL cells, CLL cells were divided into CD38-low and CD38-high groups. The CD38 high and low expression levels correlated with other prognostic markers such as cytogenetic abnormalities and IgVH mutational status in the same patient population. In the low-CD38 group, significantly decreased expression of CTLA4 was observed in CLL cells treated with AS compared to control and irrelevant AS-treated CLL cells (p<0.05). Although downregulation of CTLA4 was observed in CD38-high CLL cells, it was not significant (Figure 1C). However, down-regulation of CTLA4 significantly increased the CD38 level (p<0.05) in low CD38 CLL cells as shown in our flow cytometry results (n = 3) (Figures 1F and 1G). Further; CTLA4 downregulation was also confirmed using flow cytometry (Figures 1F and 1G). Together, these results confirm that the expression of CTLA4 was downregulated in CLL cells treated with CTLA4 AS/siRNA compared to control.

CTLA4-downregulated CLL cells were cultured to determine their proliferation rate in comparison to control CLL cells. Figure 1D and 1E show the proliferation rate of these cells as determined by both radioactive ^3^H-thymidine uptake and MTT assays using triplicates of eight different primary CLL with low CD38 samples. Cell proliferation was significantly increased in CTLA4-downregulated CLL cells compared to untreated CLL cells or to CLL cells treated with irrelevant AS (p<0.001). Overall, the proliferation rate was consistent between the three incubation times/intervals (24, 48, and 72 hours), although the highest levels of proliferation were measured in CTLA4-downregulated CLL cells incubated with AS for >48 hours. Together these results demonstrate a significant increase in proliferation in primary CLL cells with CTLA4 downregulation. Though low level of CLL cells are proliferative *in vitro*, the staining with Ki-67 revealed that CTLA4-siRNA treatment increases the Ki-67 (p<0.001) stained CLL cells, hence re-confirming its role in proliferation of CLL cells (Figure 1F and 1G).

### Upregulation of B-cell Survival/Proliferation Molecules in CTLA4-downregulated CLL Cells

**Figure 2:**
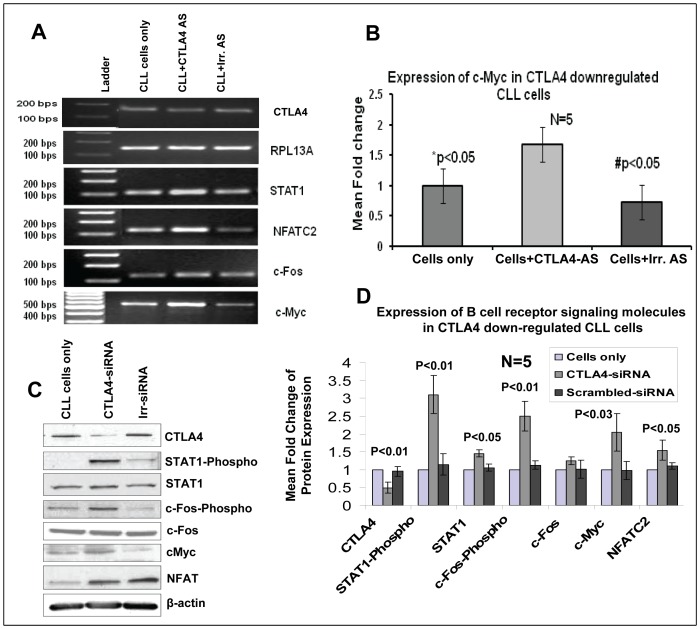
Upregulation of STAT1/phospho-STAT1, NFATC2, c-Fos/phospho-c-Fos, and c-Myc in CTLA4-downregulated CLL (low CD38/high CTLA4) cells as determined by RT-PCR, real-time PCR, and/or Western blotting. **Panels A:** Semi-quantitative RT-PCR showing that downregulation of CTLA4 by AS in CLL cells leads to up-regulation of STAT1, NFATC2, c-Fos, and c-Myc in CLL. **Panel B:** Real-time PCR results from four patient samples showing a significantly higher expression of c-Myc in CTLA4-downregulated CLL cells compared to control CLL cells or CLL cells treated with irrelevant AS. **Panel C–D:** Western blot results showing up-regulation and quantification of the expression of STAT1 and its phosphorylation, c-Fos and its phosphorylation, NFATC2, and c-Myc at the protein level in CTLA4 down-regulated CLL patient samples (n = 3). *p indicates the statistical difference between control CLL cells and CTLA4-downregulated CLL cells. β-Actin was used as a control.

To further explore the role of CTLA4 in the pathogenesis of CLL, and to confirm the involvement of CTLA4 in the regulation of the B-cell proliferation/survival signaling pathway, expression of c-Fos, phospho-c-Fos, STAT1, phospho-STAT1, NFATC2, and c-Myc was measured in control/untransfected CLL cells, CLL cells treated with irrelevant AS/siRNA, and CTLA4-downregulated CLL cells (Figure 2). Downregulation of CTLA4 in these CLL cells was confirmed by RT-PCR and western blot analyses. Furthermore, RT-PCR results showed an upregulation of STAT1, NFATC2, and c-Myc in CTLA4-downregulated CLL cells, as shown in Figure 2A. In addition, c-Myc was selected for further study because of its crucial role in cell proliferation. RT-PCR and real-time PCR results from five CLL patient samples confirmed a significant (p<0.05) upregulation of c-Myc in CTLA4-downregulated cells, as shown in Figures 2A and 2B. c-Myc expression increased by >1.5 fold in CTLA4-downregulated cells compared to control CLL cells. Further, our western blot results clearly showed that the expression levels of B-cell survival molecules including phosphorylations of STAT1 and c-Fos, STAT1, NFATC2 and c-Myc increased significantly in CTLA4 downregulated (n = 5) CLL patient samples (Figure 2C and 2D). Together, these results suggest that expression of these molecules inversely correlates with the expression of CTLA4 in CLL cells.

### Differential Expression of CTLA4 and Associated Molecules in High-CD38/Low CTLA4 and Low-CD38/High-CTLA4 CLL Groups

**Figure 3:**
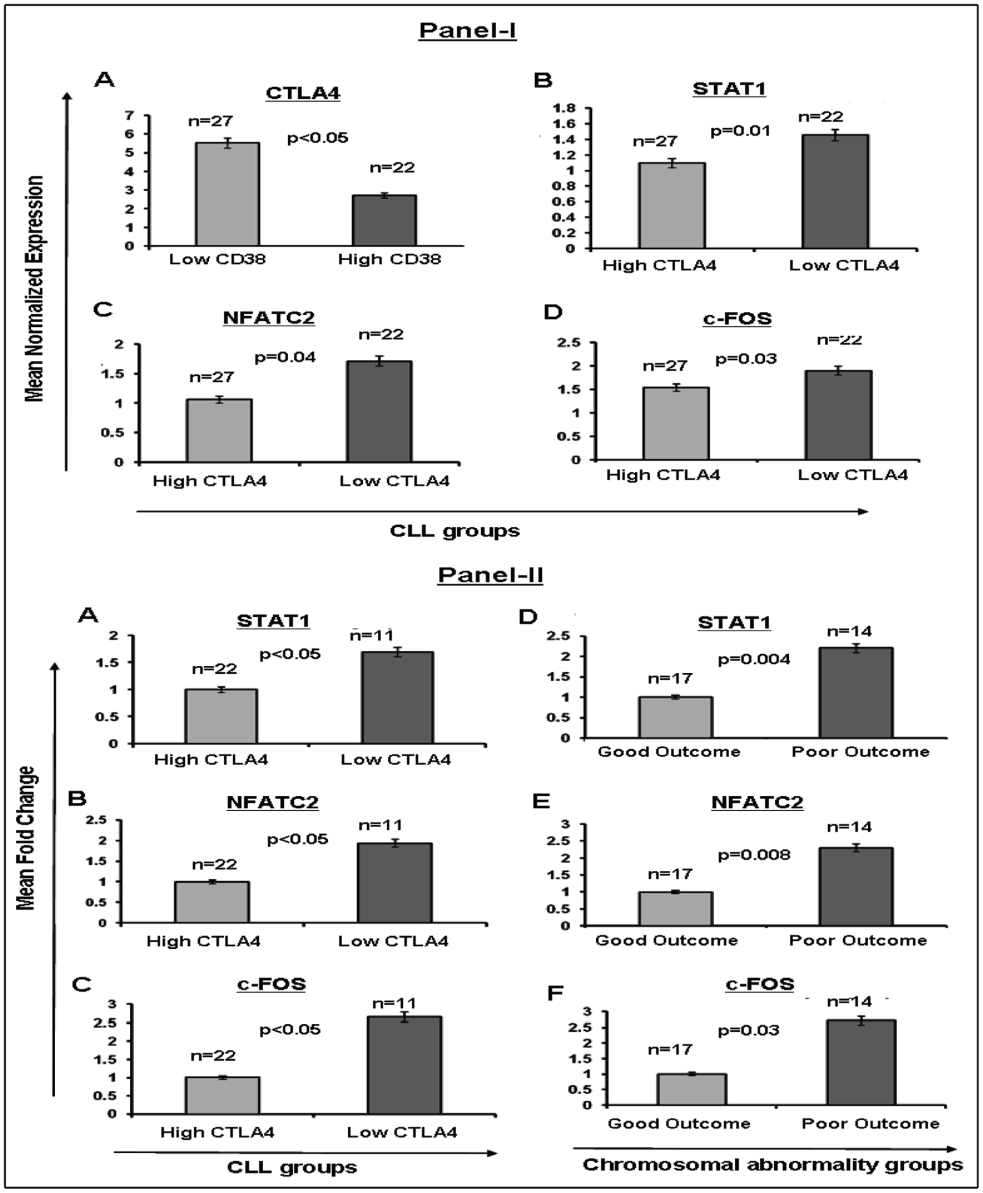
Differential expression of CTLA4 and associated molecules in primary CLL cells. **Panel I:** Differential expression of CTLA4 and associated molecules in high-CD38/low-CTLA4 and low-CD38/high-CTLA4 CLL subgroups as determined by microarray data. **Subpanel A:** Mean normalized expression levels of CTLA4 in the CD38-low group compared to the CD38-high group. **Subpanels B–D:** Mean normalized expression levels of STAT1, NFATC2, and c-Fos (respectively) in the CTLA4-high group compared to CTLA4-low group. **Panel II:** Overexpression of downstream molecules in BCR proliferation signaling in two prognostic CLL subgroups, as determined by real-time PCR. **Subpanels A–C:** Real-time PCR showing gene expression for STAT1, NFATC2, and c-Fos (fold change) in the high-CTLA4 CLL group compared to the low-CTLA4 CLL group, respectively. **Subpanels D–F:** Real-time PCR showing gene expression of STAT1, NFATC2, and c-Fos (fold change) in chromosomal abnormality subgroups, respectively (good outcome includes 13q14 deletion and normal karyotype, while poor outcome includes trisomy12, 11q deletion, and 17p deletion chromosomal abnormality).

Using microarray analysis, we previously demonstrated that CTLA4 expression inversely correlates with CD38 expression [5]. Therefore, to further explore the pathway through which CTLA4 potentially affects CLL pathogenesis, we performed microarray analyses to investigate the transcript levels of molecules associated with the BCR signaling pathway in CLL in high- and low-CTLA4 groups (Figure 3-IA). Among these molecules, STAT1 (p = 0.01) (Figure 3-IB), NFATC2 (p = 0.04) (Figure 3-IC), and c-Fos (p = 0.03) (Figure 3-ID) were found to be significantly overexpressed in low CTLA4 CLL cells. Thus, expression of these genes also inversely correlated with the expression of CTLA4 in primary CLL cells.

### Overexpression of Downstream Signaling Molecules Associated with B-cell Proliferation in Two Different Prognostic CLL Subgroups

In order to validate the microarray expression profile for the genes we selected, we performed real-time PCR on 49 different cDNA samples from CLL cells expressing either high or low CTLA4. Real-time PCR results confirmed the differential expression of STAT1, NFATC2, and c-Fos in samples from cells expressing low CTLA4 (n = 22) compared to those expressing high levels of CTLA4 (n = 27), as shown in Figure 3-II. Specifically, expression of STAT1, NFATC2, and c-Fos (Figures 3-IIA, IIB, and IIC respectively) was significantly increased (∼2 fold; p<0.05) in the low-CTLA4 CLL subgroup (or high CD38 group) in comparison to the high-CTLA4 CLL group (or low CD38 group).

Characteristic chromosomal abnormalities can serve as prognostic markers in CLL. Normal karyotype and 13q deletion are associated with good outcome, whereas 11q deletion, trisomy12, and 17p deletion are associated with poor outcome. To compare the expression of STAT1, NFATC2, and c-Fos between poor and good outcome groups (as predetermined by cytogenetic analyses), we re-analyzed the real-time PCR results based on chromosomal abnormality. Consistent with the results based on high- and low-CTLA4 expression status, significantly higher (>2 fold) expression of STAT1 (p = 0.004), NFATC2 (p = 0.008), and c-Fos (p = 0.03) was observed in the poor outcome group compared to the good outcome group (Figures 3-IID, IIE, and IIF, respectively). Together, these results confirm the activation of STAT1, NFATC2, and c-Fos in CLL cells of patients with predicted poor prognosis, whether prognosis is predicted by CTLA4/CD38 expression or by chromosomal abnormality.

### Measurement of Apoptosis in CTLA4-downregulated CLL Cells

**Figure 4:**
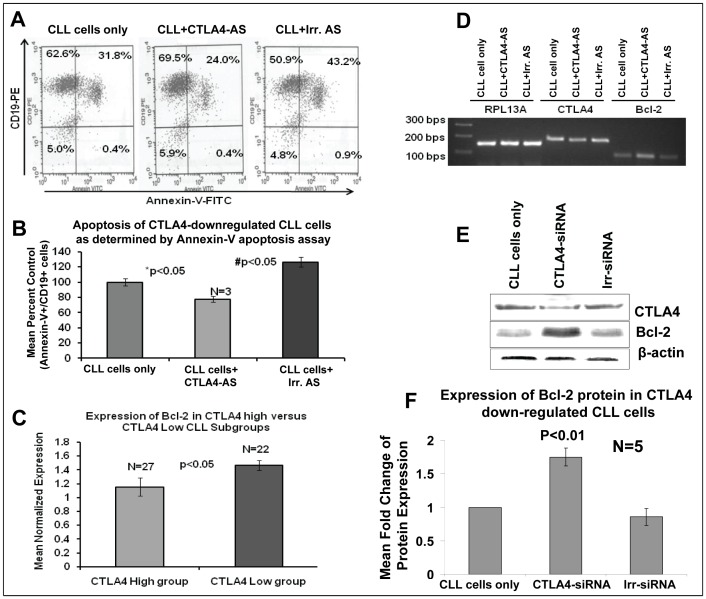
Measurement of apoptosis in CTLA4-downregulated low CD38/high CTLA4 CLL cells. **Panel A:** Representative results showing the percentage of live, pre-apoptotic, and apoptotic cells in CTLA4-downregulated CLL cells as determined by flow cytometry. **Panel B:** Mean decrease in apoptotic cell frequency in CTLA4-downregulated CLL cells. The values are derived from primary CLL cells from three different patients using Annexin-V flow cytometry method. **Panel C:** Expression of Bcl-2 in the CTLA4-high and CTLA4-low subgroups as determined by microarray. **Panels D:** Expression levels of CTLA4 and Bcl-2 in CTLA4-downregulated CLL cells as determined by semiquantitative RT-PCR. RPL13A, a housekeeping gene, was used as a control. **Panels E–F:** Bcl-2 expression levels were measured using Western blot in CTLA4-downregulated CLL cells. Panel E: A representative figure for the upregulation of Bcl-2 protein. Panel F: Mean expression of Bcl-2 in (low CD38) patient samples (n = 3) treated with and without CTLA4-siRNA. β-Actin was used as a control.

Because CLL cells often demonstrate defective apoptosis, the rate of apoptosis was measured in CLL cells with CTLA4 downregulation. CLL cells from three different patients were treated with CTLA4 AS for 72 hours. The number of B cells undergoing apoptosis was then measured in CTLA4-downregulated and control CLL cells using Annexin-V-APC and CD19-FITC staining. Flow cytometry results showed that a significant decrease in the rate of apoptosis in CTLA4-downregulated CLL (low CD38/high CTLA4) cells (Figure 4A). A representative sample is displayed in Figure 4A, which shows a decreased percentage of apoptotic cells in the CLL cell population treated with CTLA4 AS (24%) compared to the control CLL cells (31.8%) and CLL cells treated with irrelevant AS (43.2%). The mean number of apoptotic cells in each treatment group was normalized to the percent of control. The CTLA4-downregulated cell population demonstrated an apoptotic frequency of 70% compared to the control population (set at 100%, Figure 4B). This difference was significant, with p<0.05.

To further explore the role of downstream molecules regulated by CTLA4 in the survival of CLL, we focused the subsequent studies on the expression of Bcl-2, an anti-apoptotic molecule. To explore the direct relationship of CTLA4 to Bcl-2, we performed RT-PCR and western blot analyses. We observed that Bcl-2 is upregulated in CTLA4-downregulated CLL (low CD38/high CTLA4) cells at the both mRNA and protein levels, as shown in Figure 4D, 4E and 4F. Further, Bcl-2 expression increased significantly (p>0.01) in CTLA4-downregulated CLL cells (n = 5) compared to the controls (Figure 4F). Interestingly, similar to NFATC2, STAT1, and c-Fos, Bcl-2 was significantly upregulated (p<0.05) in the low CTLA4-expressing CLL group (Figure 4C). Together these results demonstrate that downregulation of CTLA4 leads to decreased apoptosis involving Bcl-2 in CLL cells.

### Influence of the Microenvironment on the Expression of CTLA4 and Associated Molecules in CLL Cells

**Figure 5:**
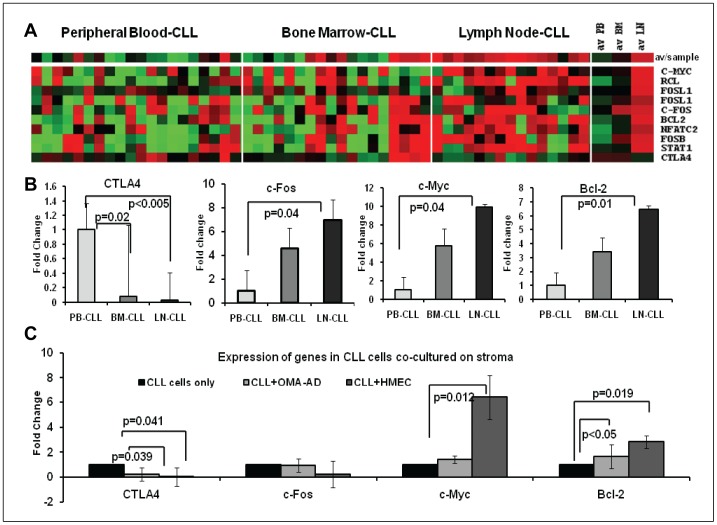
Microenvironmental influence on the expression of CTLA4 and associated molecules. **Panel A:** Supervised cluster analyses of expression of CTLA4 and associated molecules among CLL cells from three different *in vivo* microenvironments such as peripheral blood (PB-CLL, n = 20), bone marrow (BM-CLL, n = 18) and lymph nodes (LN-CLL, n = 15) using microarray analyses. **Panel B:** Validation of the differentially expressed genes among PB-CLL (n = 12), BM-CLL (n = 12), and LN-CLL (n = 12) cells using real-time PCR analyses. **Panel C:** Upregulation of CTLA4, c-Myc, and Bcl-2 in CLL cells (n = 6) co-cultured *in vitro* with stromal cells including OMA-AD and HMEC cells.

To investigate the influence of the microenvironment on the expression of CTLA4 and associated molecules, CLL cells from BM, PB, and LN of patients were isolated and processed for gene expression profiling. Interestingly, microarray analyses showed that CTLA4 was significantly underexpressed in LN-CLL cells compared to PB-CLL (p<0.005) and BM-CLL cells (p = 0.02); (Figure 5A). By contrast, NFATC2, STAT1, c-Fos, FosB, FosL1, FosL2, c-Myc, and Bcl-2 were significantly overexpressed in LN-CLL cells compared to PB-CLL and BM-CLL cells (Figure 5A). The underexpression of CTLA4 and overexpression of c-Myc, c-Fos and Bcl-2 in LN-CLL was confirmed using real-time PCR (Figure 5B). To further investigate the role of the stromal microenvironment in the induction of these genes, CLL cells (n = 6) were cultured on OMA-AD and HMEC stromal cells, and changes in gene expression were measured using real-time PCR (Figure 5C). Interestingly, CTLA4 was downregulated and c-Myc and Bcl-2 were upregulated in CLL cells grown on stroma for 72 hours compared to CLL cells cultured without stroma.

## Discussion

CD38 expression is a reliable prognostic marker: lower (<30%) expression indicates good outcome in CLL patients. However, the involvement of the signaling pathways responsible for the good outcome is not entirely studied. Previously, we proposed a hypothetical model, which predicts the pathways and genes that operate in the good outcome or low CD38-expressing CLL cells [5]. One of the key molecules proposed in the model is CTLA4, which was overexpressed in the low CD38-expressing group (indicating good prognosis). This study is focused to investigate the molecular basis of increased expression of CTLA4 on the leukemic cells of CLL patients with good prognosis.

CTLA4 is well known for its inhibitory effect on T-cell activation [24], [25]. CTLA4 can decrease the proliferation rate of T-cells by inhibiting cell cycle regulators such as cyclin-dependent kinases. However, the role of CTLA4 in the induction of apoptosis is controversial. A few studies have reported that CTLA4 can induce apoptosis [26], [27], but others report that it provides T-cell resistance to apoptosis by inducing the expression of Bcl-2 and activating the PI3K pathway [28].

Despite the number of studies unraveling the function of CTLA4 in T-cells, only a limited amount of information is known about the function of CTLA4 in B-cell or any other non-T cells. Recently, CTLA4 overexpression was also detected in non-squamous type of non-small cell lung cancer and correlated with low Ki-67 expression and reduced death rate [29]. The expression of CTLA4 on B-cells merits further investigation because there are many common ligands and receptors between B-cells and T-cells, though some are expressed dominantly on one of the cell subsets, such as the dominant expression of B7-1 and B7-2 ligands on activated B-cells [30], [31]. Within the last decade, a few key studies demonstrated a connection between the immune response and CTLA4, dramatically drawing attention to the role of CTLA4 in B-cells. One such study showed that CTLA4−/− mice produce more antibodies, indicating an active role for CTLA4 in B-cells [24], [25]. Furthermore, the expression of CTLA4 has been reported to be higher on CLL cells than on normal B-cells. The expression of CTLA4 on CLL cells predicted clinical outcome; lower expression correlated with advanced stages of disease, poor prognosis, and with high CD38 expression [5]. Recently, it was shown that CTLA4 expression on B-cells determines the early fate of B-cells in the thymus-dependent immune response [32]. Additionally, B-cells transfected with a vector coding for CTLA4-Ig expressed fewer co-stimulatory molecules on their surface [33]. Nevertheless, the functional significance of CTLA4 expression still needs to be explored.

We hypothesized that CTLA4 expressed on CLL cells inhibits their survival/proliferation by regulating the expression of downstream molecules. B-cells store significant amount of CTLA4 in intracytoplasmic vesicles. Thus, we decided to target CTLA4 at the transcript level to downregulate total CTLA4 protein in the cell. We used AS/siRNA to downregulate CTLA4 and observed up to 50% downregulation efficiency at the transcript and protein levels in low CD38-expressing CLL cells (Figure 1A–G). CD38 is a well-studied prognostic marker in CLL cells where high CD38 expression has been correlated with poor prognosis in CLL patients. CD38 expression increases the proliferation/survival of CLL cells. The inverse correlation between the expression of CD38 and CTLA4 indicated that CTLA4 may offset the proliferation/survival signals of CD38. We found a significant increase in the proliferation of CLL cells when CTLA4 was downregulated compared to controls (Figure1D and 1E). Indeed, the expression of CD38 on CLL cells was increased (Figure 1F and 1G) after treating with CTLA4-siRNA indicating a complex interaction between these two molecules. Interestingly, we found that in CTLA4-downregulated CLL cells, the expression of STAT1, NFATC2 and c-Myc is increased (Figure 2). STAT1 is a member of the family of transcription factors and forms homodimers/heterodimers with STAT2 and STAT3. STAT1 is activated in the JAK/STAT pathway, which regulates normal cell growth and survival. In CLL, interleukin 2 (IL-2) activates STAT1 and increases the proliferation of CLL cells [34]. Furthermore, activation of IL-4R and vascular endothelial growth factor receptor (VEGF-R) on CLL cells leads to activation of STAT1/STAT3 and increased survival of CLL cells [35], [36]. Expression levels of STAT1 have been correlated with a survival advantage in CLL cells [37]. In concord to these reports, we found that STAT1 and its phosphorylated form were overexpressed in CTLA4-downregulated cells. Because these cells also showed a significant increase in proliferation and survival compared to control, we can infer that CTLA4 modulates survival/proliferation of CLL cells via regulating the JAK/STAT pathway.

NFAT molecules have been previously studied in CLL cells, and increased transactivation of NFAT has recently been reported in CLL [38], [39]. NFATC2 binds to the promoter of CTLA4 and controls its expression [40]. We found that expression of NFATC2 was increased when CTLA4 was down-regulated, which indicates a potential feedback loop between CTLA4 and NFATC2. This possibility requires future study. Additionally, c-Fos was found to be upregulated in the high-CD38 CLL group and in CTLA4-downregulated CLL cells. Members of the Fos family contain a leucine zipper and are able to dimerize with the proteins of the JUN family [41], [42]. The role of c-Fos is very well studied in the regulation of cell proliferation, differentiation, and survival [43], [44]. Upregulation of c-Fos has already been reported in unmutated-IgVH or poor-prognosis CLL subgroup, and the activation of c-Fos has been reported in CLL cells undergoing invasion and migration [45], [46]. In the current study, we found c-Fos phosphorylation to be upregulated in the CTLA4-downregulated CLL cells with increased proliferation, suggesting that CTLA4 inhibits proliferation in part by regulating the activation of c-Fos.

Although c-Myc was not differentially expressed between high- and low-CD38 subgroups, it was included in our study because: it is a transcription factor that plays a critical role in certain cancers [47]. c-Myc is commonly involved in the transformation and proliferation of cells, and it has been shown to induce the growth of CLL cells [47], [48]. We found c-Myc to be significantly upregulated in CLL cells with CTLA4 downregulation (Figure 2A and 2C). These results indicate that CTLA4 may control the expression of c-Myc, but further studies are needed to confirm a direct relationship between them.

CLL cells are known for their inherent resistance to apoptosis. Thus, after examining the role of CTLA4 in proliferation, we studied its influence on the apoptosis of CLL cells. Patients with CLL cells that express higher levels of CD38 tend to show aggressive disease than those with low levels of CD38 [3]. Considering the inverse relationship between CTLA4 and CD38 expression in CLL cells, it was reasonable to think that CTLA4 expression promotes apoptosis in CLL cells. We examined the expression of Bcl-2 in CLL because it is well-known for its role in inhibiting apoptosis in CLL cells [48], [49]. As expected, CTLA4-downregulated CLL cells showed increased survival and increased expression of Bcl-2 in our study (Figure 4). These results indicate that CTLA4 regulates Bcl-2 expression in CLL cells. However, we wanted to know whether CTLA4 expression regulates STAT1, NFATC2, c-Fos, and Bcl-2 *in vivo*. To this end, we divided the CLL patient samples into either high-CTLA4 (low-CD38) or low-CTLA4 (high-CD38) groups and measured the expression of these genes using microarray analyses. We found differential gene expression between these two groups (Figure 3). Moreover, expression of these genes was higher in the CLL group with chromosomal abnormalities associated with poor prognosis, indicating that these gene products might increase the risk of aggressive disease. Thus, our observations suggest that CTLA4 regulates the expression/activation of STAT1, NFATC2, c-Fos, and Bcl-2 in disease contexts.

It is well accepted that the microenvironment can influence the survival and proliferation of CLL cells [50]. The microenvironment of BM and LN stimulates CLL cell proliferation [51]–[53]. Interestingly, the expression of CD38 is also regulated by the microenvironment, and it was found that CD38 is highly expressed on LN-CLL [54]. Thus, we investigated the expression of CTLA4 in CLL cells harvested from an *in vivo* microenvironment such as the lymph node. As expected, we observed an inverse correlation between CD38 and CTLA4 expression in CLL cells from lymph node (Figure 5). CTLA4 was significantly less expressed in LN-CLL cells. Furthermore, downstream signaling molecules including NFATC2, STAT1, c-Fos, c-Myc, and Bcl-2 were significantly overexpressed in LN-CLL cells compared to PB-CLL and BM-CLL cells. Furthermore, to validate that stroma can induce gene expression, we tested two stromal systems. Interestingly, CLL cells co-cultured on stroma showed down-regulation of CTLA4 and upregulation of c-Fos and Bcl-2. These observations indicate that the microenvironments can downregulate the expression of genes that cause cell death and inhibition of cell proliferation, such as CTLA4. This would indirectly upregulate the genes related to proliferation/survival, such as c-Fos, c-Myc, and Bcl-2.

**Figure 6:**
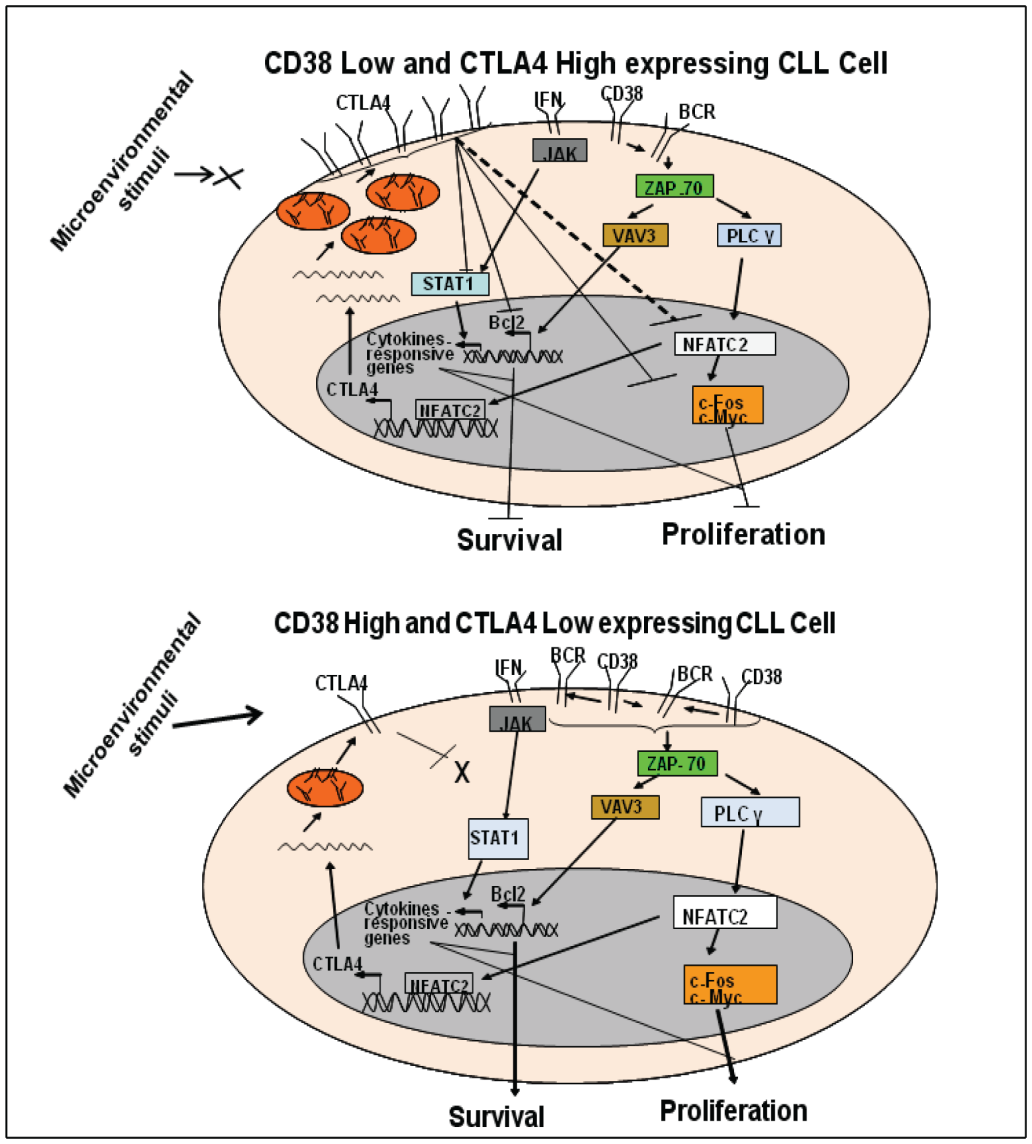
Hypothetical model for the role of CTLA4 in CLL cell proliferation/survival. **Panel A:** When CLL cells express low CD38, but high CTLA4, CTLA4 inhibits the CD38/BCR signaling pathway at multiple levels. CTLA4 downregulates NFATC2 and proliferation-associated molecules such as c-Fos and c-Myc. Downregulation of NFATC2 may also be associated with an autoregulatory loop for CTLA4, which would downregulate CTLA4 transcription. CTLA4 also downregulates the expression of Bcl-2, thus decreasing the survival of CLL cells. CTLA4 inhibits the expression of STAT1, thus deregulating the JAK/STAT pathway and inhibiting CLL cell growth. **Panel B:** When CLL cells express high CD38, but low CTLA4, activated CD38/BCR signaling upregulates downstream molecules in the pathway, such as NFATC2, c-Fos, and Bcl-2. These molecules will increase proliferation and survival of CLL cells. Low expression of CTLA4 does not interfere with the expression of STAT1, which favors CLL cell growth.

Taken together, our study shows that CTLA4 inhibits the proliferation/survival of CLL cells via regulation of the expression of STAT1, NFATC2, c-Fos, c-Myc and Bcl-2. A hypothetical model for CTLA4-mediated regulation of CLL cell proliferation/survival is shown in Figure 6. In this model, we propose that the expression of CTLA4 is governed by the microenvironment. The tumor microenvironment in CLL suppresses the expression of CTLA4. CTLA4 inhibits the B-cell proliferation/survival pathway at multiple levels by down-regulating key downstream molecules including transcription factors STAT1 and NFATC2, proliferation-associated c-Myc, and anti-apoptotic Bcl-2, resulting in decreased proliferation/survival of CLL cells (Figure 6). Therefore, by controlling CTLA4 expression, manipulation of proliferation/survival of CLL cells is feasible, which offers a new avenue to improve therapy for CLL.

## Supporting Information

Table S1 Patients’ Characteristics. Peripheral blood samples from 105 CLL patients were screened for the CD38 prognostic marker. Multi-variant analysis of CLL subgroups based on high (>30% positive cells) and low (<30% positive cells) percentages of the CD38 marker were correlated with other known prognostic markers. (DOCX)

Table S2 List of primers and sequences of oligonucleotide antisense RNA (F = Forward, R = Reverse). (DOCX)
