# Supplementary material for: Role of CTLA4 in the Proliferation and Survival of Chronic Lymphocytic Leukemia
Source: PLoS One. 2013 Aug 1;8(8):e70352. doi: 10.1371/journal.pone.0070352 (PMC3731360; doi:10.1371/journal.pone.0070352)
Supplement: Table S1 — (DOCX) [file pone.0070352.s001.docx]

**Supplementary Table I: Patients’ Characteristics.** Peripheral blood samples from 105 CLL patients were screened for the CD38 prognostic marker. Multi-variant analysis of CLL subgroups based on high (>30% positive cells) and low (<30% positive cells) percentages of the CD38 marker were correlated with other known prognostic markers.

| **Parameters** | **High CD38**  **(>30%), n=37** | **Low CD38 (<30%), n=68** | **p-value** |
| --- | --- | --- | --- |
| **Gender**  Males  Females | 66.6%  33.3% | 62.6%  37.3% | NA |
| **Age (years)**  Mean  Median | 62.5  61.5 | 60.4  61 | NA |
| **% of CD38+ cells**  Mean  Median | 55%  52% | 11.3%  9% | <0.0001 |
| **Mutational status**  Unmutated  Mutated | (N=13)  84.6%  15.3% | (N=17)  32.3%  64.7% | NA |
| **Chromosomal abnormalities**  13q14 del.  Normal karyotype  Trisomy12  11q22  17p del. | 11.4%  28.5%  31.4%  22.8%  0.05% | 60.2%  22.4%  10.3%  6%  None | NA |
| **Beta-2-microglobulin**  Mean  Median | 3.65  3.0 | 2.3  2.2 | 0.007 |
| **Rai Stages**  0  1  2  3  4 | (N=24)  11.11%  22.22%  16.66%  5.55%  44.44% | (N=39)  56.41%  20.51%  2.56%  5.12%  15.38% | NA |
| **Lymphadenopathy**  Present  Absent | (N=24)  79.16%  20.83% | (N=37)  29.72%  70.27% | NA |
| **Patients Therapy**  Treated  Untreated | 67.74%  32.25% | 28%  72% | NA |
