# Supplementary material for: Role of CTLA4 in the Proliferation and Survival of Chronic Lymphocytic Leukemia
Source: PLoS One. 2013 Aug 1;8(8):e70352. doi: 10.1371/journal.pone.0070352 (PMC3731360; doi:10.1371/journal.pone.0070352)
Supplement: Table S2 — (DOCX) [file pone.0070352.s002.docx]

**Supplementary Table II:** List of primers and sequences of oligonucleotide antisense RNA (F = Forward, R = Reverse).

| **Name of gene** | **Genebank accession no.** | **Sequence**  **5’ ----> 3’** | **Tm °C** | **Prod-uct size** |
| --- | --- | --- | --- | --- |
| HPRT-F  HPRT-R | NM_000194 | AGGGTGTTTATTCCTCATGGAC  GTAATCCAGCAGGTCAGCAAAG | 60 | 103 |
| RPL13A (F)  RPL13A (R) | NM_012423 | ACCGTCTCAAGGTGTTTGACG  GTACTTCCAGCCAACCTCGTG | 58 | 125 |
| Fos (F)  Fos (R) | NM_005252 | TGGCGTTGTGAAGACCATGACAG  GCTGCAGCCATCTTATTCCTTTCC | 58 | 104 |
| NFATC2- (F)  NFATC2- (R) | NM_012340 | CCAAGACGAGCTTGACTTCTCCAT  ATCATCGGGGTATGCGGGTC | 58 | 118 |
| Bcl2 (F)  Bcl2 (R) | M13994 | GCATGCGGCCTCTGTTTGATTTCT  AGGCATGTTGACTTCACTTGTGGC | 60 | 140 |
| c-Myc (F)  c-Myc (R) | NM_002467 | TTTCCCAGAAAAGCCATTCC  GCCGAAGTAGAAGTCATCTTC | 60 | 340 |
| STAT1- (F)  STAT1-(R) | NM_007315 | TCATCTTCTCTGGCGACAGTTTTC  GCGAATTTGCTGGCCTTTCT | 58 | 109 |
| CTLA4- (F)  CTLA4- (R) | NM_005214 | TGGCTTGCCTTGGATTTCAGC  ACACACAAAGCTGGCGATGC | 62 | 173 |
| CTLA4 Antisense (AS) | NM_005214 | ATGGCTTGCCTTGGATTTCA |  |  |
| Irrelevant Antisense (AS) |  | TATGCTGTGCCGGGGTCTTCGGGC |  |  |
